# Focused Ultrasound-Mediated Blood–Brain Barrier Opening for Enhanced Drug Delivery in Diffuse Gliomas: A Systematic Review

**DOI:** 10.3390/biomedicines14102251

**Published:** 2026-10-05

**Authors:** Gabriele Ciaffi, Renata Martinelli, Andrea Traverso, Benedetta Burattini, Placido Bruzzaniti, Fabio Papacci, Andrea Talacchi, Giuseppe Maria della Pepa, Giovanni Pennisi

**Affiliations:** 1 Department of Neurosurgery, Fondazione Policlinico Universitario A. Gemelli IRCCS, 00168 Rome, Italy; gabriele.ciaffi@hotmail.it (G.C.); martinellirenata97@gmail.com (R.M.); andrea.traverso@guest.policlinicogemelli.it (A.T.); benedetta.burattini@gmail.com (B.B.); fabio.papacci@policlinicogemelli.it (F.P.); giuseppemria.dellapepa@policlinicogemelli.it (G.M.d.P.); 2 Department of Neurosurgery, San Carlo Borromeo Hospital, 20153 Milan, Italy; placntz@gmail.com; 3 Azienda Ospedaliera San Giovanni Addolorata, Neurochirurgia, 00184 Rome, Italy; atalacchi@hsangiovanni.roma.it

**Keywords:** focused ultrasound, blood–brain barrier opening, glioma, glioblastoma, drug delivery, microbubbles, MRgFUS, systematic review, neuro-oncology

## Abstract

**Background/Objectives:** The blood–brain barrier (BBB) severely limits the intratumoral delivery of systemically administered therapeutics in diffuse gliomas, including glioblastoma (GBM), contributing to the persistently poor prognosis of these tumors. Focused ultrasound (FUS) combined with microbubble agents enables transient, spatially targeted, and reversible BBB opening (BBBO), offering a non-invasive strategy to enhance drug delivery in this setting. Despite a rapidly growing body of clinical evidence, a systematic synthesis of outcomes across FUS platforms, drug regimens, and study designs is currently lacking. This systematic review aims to critically appraise the available clinical evidence on FUS-mediated BBBO for enhanced drug delivery in adult patients with diffuse gliomas. **Methods:** A systematic search of PubMed/MEDLINE and Scopus was performed using pre-specified search terms (last search: June 2026). Eligible studies included clinical trials and cohort studies enrolling adult patients (≥18 years) with histologically confirmed diffuse glioma (WHO grades II–IV) who underwent FUS-BBBO for therapeutic drug delivery enhancement. Two reviewers independently screened records and extracted data using a standardized form. Study quality was assessed using the NIH Quality Assessment Tool for Before-After Studies (single-arm studies) and the ROBINS-I tool (comparative studies). The review was retrospectively registered on OSF. Results were synthesized narratively due to study heterogeneity. **Results:** Eight publications from seven independent clinical studies were included. Across these publications, 68 patient entries were reported; however, because of overlapping populations across publications, the number of unique patients could not be precisely determined. Two FUS platforms were employed, with the ExAblate Neuro system (220–230 kHz) used in six out of seven cohorts. BBB opening was confirmed in all studies, with target sonication success rates of 92.4–100%. No procedure-related serious adverse events attributable to FUS-BBBO were reported across any study. Transient T2/SWAN hypointense foci were observed in a subset of patients, consistently asymptomatic and histologically benign. Direct intratumoral drug quantification was feasible in two pre-surgical studies, demonstrating a 2.2-fold increase in drug accumulation in FUS-treated versus untreated tissue (*p* < 0.01). The only comparative study reported a statistically significant improvement in overall survival (median 31.3 vs. 19.3 months; HR 0.50, *p* = 0.009) and progression-free survival (median 13.5 vs. 8.1 months; HR 0.55, *p* = 0.020) versus the controls. **Conclusions:** FUS-mediated BBBO is a safe and feasible approach for enhancing drug delivery in adult diffuse glioma patients across multiple platforms and drug combinations. Preliminary comparative data suggest a potential survival benefit in newly diagnosed GBM; however, these findings are based on a single non-randomized study and require confirmation in prospective randomized controlled trials.

## 1. Introduction

Diffuse gliomas represent the most common primary malignant brain tumors in adults, with glioblastoma (GBM, WHO grade IV) accounting for approximately 50% of all primary malignant brain tumors and carrying a median survival of 14–16 months despite maximal safe resection, concurrent radiotherapy, and temozolomide chemotherapy according to the Stupp protocol [1,2]. Lower-grade gliomas (WHO grades II–III) follow a more indolent course but invariably progress, with a subset undergoing malignant transformation to higher-grade disease [3,4]. The persistently dismal prognosis of GBM, largely unchanged over the past two decades, underscores the urgent need for novel strategies to enhance therapeutic efficacy [5,6,7].

A principal obstacle to effective pharmacological treatment of diffuse gliomas is the blood–brain barrier (BBB), a highly specialized neurovascular unit that tightly regulates molecular trafficking between the systemic circulation and the central nervous system [8]. Although the BBB is partially disrupted within the enhancing core of high-grade gliomas, it remains largely intact in the infiltrative peritumoral regions where invasive tumor cells reside and resist surgical removal [9,10]. Critically, carboplatin and paclitaxel concentrations in peritumoral tissue have been reported to be up to 40-fold lower than at the tumor centre, rendering systemically administered agents largely ineffective against diffusely infiltrating disease [11]. Strategies to overcome the BBB have historically carried significant limitations: convection-enhanced delivery requires invasive neurosurgical access, receptor-mediated transcytosis approaches demonstrate limited spatial specificity and off-target effects, and radiation-induced permeability is unpredictable and delayed [11,12,13]. Focused ultrasound (FUS) combined with intravenously administered microbubble agents has emerged as a promising non-invasive strategy to achieve targeted, transient, and reversible BBB opening (BBBO) [14]. When circulating microbubbles interact with a focused acoustic energy field, they undergo stable oscillations that mechanically disrupt the tight junctions of cerebrovascular endothelial cells, transiently increasing BBB permeability without inducing irreversible tissue damage [15]. The magnitude and spatial distribution of BBB opening can be modulated in real-time through acoustic emissions monitoring (AEM), which detects the harmonic signatures of microbubble cavitation and enables closed-feedback loop control of the procedure [16,17,18]. Three main device categories are currently available for clinical application: stereotactic frame-based MRI-guided systems (MRgFUS; e.g., ExAblate Neuro, InSightec), implantable transducer systems (e.g., SonoCloud, CarThera), and frameless neuronavigation-guided devices (e.g., NaviFUS) [13,19]. Each platform employs distinct technical approaches, yet all share the fundamental mechanism of microbubble-mediated BBBO, enabling the enhanced delivery of chemotherapeutic agents, monoclonal antibodies, nanoparticles, and immunotherapeutics into CNS tumor tissue [14,20] (Figure 1).

Preclinical evidence for FUS-mediated drug delivery enhancement in glioma models has been accumulating for over two decades, demonstrating improved concentrations of temozolomide, liposomal doxorubicin, and carboplatin in sonicated brain tissue, as well as prolonged survival in rodent glioma models [18,21,22,23,24]. Translation to clinical trials progressed rapidly following the first-in-human report of MRgFUS BBBO in primary brain tumors [11,25], with subsequent studies demonstrating not only the safety and feasibility of repeated BBBO across multiple cycles, but most recently, a statistically significant improvement in overall survival in a multicentre cohort of newly diagnosed glioblastoma patients [26]. The field has simultaneously expanded to include novel therapeutic combinations such as FUS combined with re-irradiation, and emerging applications such as sono-liquid biopsy through enrichment of circulating tumor DNA [20,26]. Despite this progress, a comprehensive synthesis of the available clinical evidence, encompassing drug delivery quantification, oncological outcomes, safety profiles, and technical optimization across different FUS platforms, remains lacking. The aim of this systematic review is to critically appraise the clinical evidence on FUS-mediated BBBO for enhanced drug delivery in adult diffuse gliomas, with the objective of informing both current clinical practice and the design of future trials.

## 2. Methods

This systematic review was conducted in accordance with the PRISMA 2020 (Preferred Reporting Items for Systematic Reviews and Meta-Analyses) framework to ensure a rigorous and transparent identification, screening, and critical appraisal of the available clinical evidence [27]. The PRISMA-SR flow diagram is presented in Figure 2. Although no formal meta-analysis was performed owing to the limited number of available studies and the substantial heterogeneity in patient populations, FUS systems, drug regimens, and outcome measures, the methodological approach followed the principles outlined in the Cochrane Handbook for Systematic Reviews of Interventions (version 6.3) [28].

The research question was formulated according to the PICO (Population, Intervention, Comparator, Outcomes) framework as follows: “In adult patients (≥18 years) with histologically confirmed diffuse glioma (WHO grades II–IV) (P), does the use of focused ultrasound (FUS) for blood–brain barrier opening (BBBO) (I), when available compared to standard of care without FUS (C), improve intratumoral drug delivery and/or clinical outcomes including overall survival, progression-free survival, and safety profile (O)?”. In the absence of a comparator arm, single-arm studies reporting at least one drug delivery or clinical safety outcome were considered eligible as preliminary evidence.

A systematic search of electronic databases (PubMed, Scopus) was performed using the following search terms: *(“focused ultrasound” OR “FUS” OR “MRgFUS” OR “MR-guided focused ultrasound” OR “low-intensity focused ultrasound” OR “LIFU” OR “transcranial focused ultrasound”) AND (“blood-brain barrier” OR “BBB” OR “blood brain barrier opening” OR “BBB disruption” OR “BBB permeability”) AND (“drug delivery” OR “chemotherapy delivery” OR “therapeutic delivery” OR “drug transport” OR “pharmacological delivery”) AND (“glioma” OR “glioblastoma” OR “astrocytoma” OR “oligodendroglioma” OR “diffuse glioma” OR “high-grade glioma” OR “low-grade glioma” OR “GBM”)*. The most recent search was conducted in June 2026. Two authors (R.M.; G.C.) independently screened abstracts for eligibility, while a senior author (G.P.) conducted a secondary review of the selected articles.

Studies were included if they met all of the following criteria: (1) conducted in adult patients with a histologically confirmed diagnosis of diffuse glioma (WHO grades II–IV), encompassing astrocytoma, oligodendroglioma, and glioblastoma; (2) FUS was employed specifically for BBB opening, with the aim of enhancing the local delivery of a co-administered treatment modality, including pharmacological agents or radiation therapy; (3) the administered agent, including pharmacological tracers and surrogate markers used to assess BBB permeability, and (4) at least one clinical or safety outcome was reported. Studies were excluded if they involved pediatric populations (<18 years); studies employing implantable devices, reported exclusively preclinical data; used FUS solely for thermal ablation without BBBO; and involved non-glial brain tumors. Review articles, editorials, letters to the editor, conference abstracts, and case reports were also excluded. Studies employing implantable ultrasound devices requiring craniotomy (e.g., SonoCloud-9, CarThera) were also excluded, as these systems represent a distinct technical class—surgically implanted transducers—not directly comparable to the transcranial or neuronavigation-guided external FUS platforms examined in this review.

Given the marked heterogeneity in study design across eligible publications, two validated tools were applied according to the design of each study, and quality assessment was performed at the level of individual publications rather than study cohorts, as each article was considered to represent a distinct analytical and methodological contribution regardless of patient overlap. For single-arm studies, the *NIH Study Quality Assessment Tool for Before-After (Pre-Post) Studies Without Control Group* was used [29]. For the only study incorporating a comparative arm—Woodworth et al. 2025 (BT008NA/ReFOCUSED; S2)—the *ROBINS-I (Risk Of Bias In Non-randomised Studies of Interventions)* tool was applied [30].

Quality assessment was performed independently by two reviewers (R.M., G.C.); discrepancies were resolved by discussion with a senior co-author (G.P.). Results are presented as a colour-coded heatmap (NIH tool) and a structured domain table (ROBINS-I) in the Supplementary Materials (Figures S1 and S2). The review protocol was registered on the Open Science Framework (registration DOI: 10.17605/OSF.IO/B5YWC).

## 3. Results

### 3.1. Study Selection

The systematic search retrieved 399 records (PubMed: 144; Scopus: 255); after the removal of 129 duplicates and title/abstract screening, 10 full-text articles were assessed for eligibility, of which 8 publications from 7 independent clinical studies were included in the final review. Two articles were excluded at full-text review: one conference abstract and one preclinical animal study. Two publications derived from the same 6-patient cohort (NCT03712293) and were treated as a single study with progressive follow-up reporting [31,32]; two further publications [13,18] represented technical substudies of the multicentre BT008 trial, whose primary efficacy results are reported by Woodworth et al. [26]. Patient counts and survival data were not duplicated across overlapping publications. The PRISMA 2020 flow diagram is provided in Figure 2.

Regarding the risk of bias assessment, seven single-arm studies were evaluated using the NIH Quality Assessment Tool: six received a Fair overall rating, reflecting limitations inherent to early-phase feasibility designs (absence of sample size justification, no blinding, no control arm); Chen et al. was rated Poor owing to the interim analysis and incomplete follow-up [20]. Woodworth et al. was assessed with ROBINS-I and received a Moderate risk of bias rating, with residual confounding from the non-randomized design as the primary concern [26]. Detailed domain-level assessments are provided in the Supplementary Materials (Figures S1 and S2).

### 3.2. Study Characteristics and Patient Population

Included studies spanned from 2019 to 2026 and originated from South Korea, Canada, the United States, Taiwan, and Italy. Study designs comprised one Phase II non-randomized controlled trial, two Phase I feasibility studies, one Phase 0 window-of-opportunity study, one retrospective technical analysis, one pilot trial with interim analysis, and one technical case series. Across the 7 independent cohorts, a total of 68 patient entries were recorded. The exact number of unique individuals cannot be precisely determined from the published data, as partial patient overlap exists between the multicentre BT008 trial [26] and its institutional substudies [13,18]; patient counts and survival data have not been duplicated in this review.

The majority of patients had glioblastoma (WHO grade IV, IDH wild-type), with the exception of one study exclusively enrolling WHO grades II–III IDH-mutant infiltrating gliomas. Mean patient age ranged from 32.5 to 55.8 years across studies. Treatment lines were heterogeneous: four cohorts were treated in the adjuvant post-chemoradiotherapy setting, two in the neoadjuvant pre-surgical setting, and one in the recurrent disease setting with re-irradiation. A detailed summary of the study characteristics and patient demographics is provided in Table 1.

### 3.3. FUS Systems and Technical Parameters

Two FUS platforms were employed across the included studies. The ExAblate Neuro system (InSightec, 220–230 kHz) was used in six out of seven cohorts, representing the predominant technology in the current clinical literature. The NaviFUS system (500 kHz), a frameless neuronavigation-guided device, was employed in one study. Definity microbubbles (perfluorocarbon, Lantheus) were used in all ExAblate-based protocols, administered as intravenous bolus or continuous infusion; SonoVue was used in the NaviFUS study. The number of treatment sessions ranged from a single pre-surgical session to up to 6 monthly cycles in the adjuvant setting. Reported acoustic power ranged from 3.4 to 30 W across studies; acoustic pressure values were not consistently reported. Full technical parameters are detailed in Table 2.

### 3.4. Blood–Brain Barrier Opening

Successful BBB opening was confirmed in all included studies, verified in every case by post-sonication gadolinium-enhanced T1-weighted MRI. Target sonication success rates ranged from 92.4% to 100%. Where quantified, the extent of opening correlated positively with acoustic emissions-derived cavitation dose, supporting the role of real-time acoustic monitoring as a tool for procedural control. The mean treated brain volume in the largest technical series was 20.5 ± 4.63 cc. Reversibility was confirmed in all studies where it was formally assessed, with the resolution of contrast enhancement within 20–24 h of the procedure.

### 3.5. Drug Delivery Efficacy

Direct quantification of intratumoral drug concentrations was feasible in two pre-surgical studies, both exploiting the window of opportunity between FUS delivery and tumor resection. Higher drug concentrations in sonicated versus non-sonicated peritumoral tissue were demonstrated by LC-MS in one study [11], though quantitative conclusions were limited by small tissue sample volumes. More robust evidence was provided by Anastasiadis et al. [33], who demonstrated a 2.2-fold increase in fluorescein accumulation in FUS-treated versus untreated non-enhancing tumor tissue (*p* < 0.01), with comparable vascular density across regions, directly implicating BBB permeability rather than vascular differences as the underlying mechanism. In the remaining studies, drug delivery enhancement was inferred indirectly from radiological confirmation of BBB opening. Woodworth et al. [26]. additionally reported the first clinical sono-liquid biopsy data, demonstrating enrichment of circulating tumor DNA (cfDNA) following FUS-BBBO.

### 3.6. Clinical Outcomes

Comparative survival data were available from a single study, the only one to include a control arm [26]. In this Phase II multicentre trial (n = 34 newly diagnosed GBM), FUS-BBBO combined with adjuvant TMZ was associated with a statistically significant improvement in both overall survival (median 31.3 vs. 19.3 months; HR 0.50, *p* = 0.009) and progression-free survival (median 13.5 vs. 8.1 months; HR 0.55, *p* = 0.020) compared to the controls. In the single-arm Korean cohort [31,32], all 6 patients were alive at 1-year follow-up, with two recurrences occurring at 11 and 16 months. Among the recurrent HGG patients treated with FUS plus re-irradiation [20], the objective response rate was 16.7% (1 partial response, 2 stable disease), with a median PFS of 97.5 days. Formal radiological response assessment was not reported in the pre-surgical or technical studies. No quality of life data were reported in any included study. Drug delivery efficacy and clinical outcomes are summarized in Table 3.

### 3.7. Safety Profile

FUS-related serious adverse events (SAE) were reported across any of the included studies. In the largest comparative cohort 87 FUS-related adverse events were recorded across all treatment cycles: 40 grade 1, 46 grade 2, and 1 grade 3 (headache attributable to the stereotactic frame, not to FUS-BBBO itself). No grade 4 or 5 FUS-related events occurred [26]. The most common imaging finding was the appearance of transient T2*/SWAN hypointense foci (petechiae) within the sonicated regions, observed in two studies [13,18]. These were consistently asymptomatic and resolved spontaneously within weeks; histological analysis in surgically resected tissue confirmed the absence of true microhemorrhages, with findings attributed to transient microleakage or venular dilatation. New GRE/T2* signal changes were also observed in approximately 50% of targeted subspots in one pre-surgical study [33], again without a histopathological correlate. One FUS-related grade 3 AE was reported by Woodworth et al. [26] (headache following frame placement). Grade 3 or higher adverse events reported across studies were attributable to concurrent chemotherapy, surgical complications, or re-irradiation-related effects, and were not considered FUS-related. Complete data regarding safety profiles are available in Table 4.

## 4. Discussion

### 4.1. Principal Findings

This review synthesizes clinical evidence on FUS-mediated BBBO for drug delivery in 8 publications from seven independent clinical studies, reporting a total of 68 patient entries across publications. Three consistent findings emerged: BBB opening was achieved in all studies across both FUS platforms employed; the procedure was uniformly well-tolerated, with no FUS-attributable serious adverse events; and the only comparative study reported a statistically significant survival benefit over the controls [26]. These findings collectively support the biological plausibility and preliminary clinical promise of FUS-BBBO while highlighting the need for prospective randomized confirmation.

### 4.2. FUS-BBBO as a Drug Delivery Strategy

Direct quantification of intratumoral drug concentrations was feasible only in the two pre-surgical window-of-opportunity studies. Anastasiadis et al. [33] provided the most rigorous evidence, demonstrating a 2.2-fold increase in fluorescein accumulation in FUS-treated versus untreated tumor tissue (*p* < 0.01), with equivalent vascular density across regions, directly implicating BBB permeability rather than tumor vascularity as the operative mechanism. Mainprize et al. [11] observed a similar trend for TMZ and liposomal doxorubicin by LC-MS, though quantitative conclusions were limited by small tissue volumes. In the adjuvant studies, drug delivery enhancement was inferred indirectly from the survival outcomes, as tissue sampling is not feasible in this setting. Developing validated non-invasive surrogates for intratumoral drug quantification, such as dynamic contrast-enhanced MRI or radiolabelled tracers, remains a key methodological priority [8]. Radiolabelled tracers and radioactive compounds represent an additional non-invasive modality for quantifying intratumoral drug delivery; PET-based approaches could enable the spatial mapping of tracer distribution relative to sonicated brain regions, providing a quantitative and clinically applicable measure of BBBO efficacy [34,35].

### 4.3. Clinical Efficacy: A Critical Appraisal

The survival benefit reported by Woodworth et al. (median OS 31.3 versus 19.3 months (HR 0.50, *p* = 0.009) and median PFS 13.5 versus 8.1 months (HR 0.55, *p* = 0.020) is the most clinically significant finding in the FUS-BBBO literature to date, particularly in the context of GBM where the Stupp protocol has remained the standard of care for two decades without meaningful improvement [2]. However, the non-randomized design with a historical control cohort, incomplete reporting of baseline prognostic variables (including MGMT methylation status), and the moderate ROBINS-I risk of bias rating all preclude a definitive causal interpretation. A Phase III RCT is necessary, and based on these data, scientifically justified. The remaining single-arm studies, reporting 100% one-year survival in the cohort evaluated by Park et al. [31,32] and an ORR of 16.7% in recurrent HGG with re-irradiation [20], contribute hypothesis-generating rather than efficacy evidence.

### 4.4. Safety Profile

The absence of FUS-BBBO-attributable serious adverse events across all studies is the most robust and reproducible finding in this review. The most frequent imaging observation of transient T2*/SWAN hypointense foci within the sonicated regions was consistently asymptomatic and self-resolving. Histological analysis in surgically resected FUS-treated tissue, reported by both Anastasiadis et al. [33] and Del Bene et al. [13], found no evidence of microhaemorrhage or tissue damage, supporting their attribution to transient microleakage or venular dilatation rather than true haemorrhagic injury. Crucially, McDannold et al. [18] demonstrated that protocol optimization, specifically, reducing the cavitation dose goal from −6 to −9 dB and extending individual sonication duration from 60 to 90 s, substantially reduced petechiae incidence while preserving BBB opening efficacy, illustrating that the therapeutic window can be widened through procedural refinement.

### 4.5. Technical Heterogeneity and Standardization

Cross-study comparison is substantially limited by heterogeneity in FUS platforms, acoustic parameters, microbubble protocols, and post-procedural imaging. Acoustic power was reported in inconsistent units (watts versus mechanical index), subsonication target numbers and spacing varied widely, and the adoption of SWAN sequences for vascular damage assessment was not uniform. Del Bene et al. [13] contributed a relevant technical advance with an optimized in-transducer MRI protocol that reduces the total procedure time from 5 to 3 h without compromising imaging quality, demonstrating that workflow innovation can meaningfully improve clinical feasibility. The choice of operating frequency represents a further axis of technical heterogeneity: lower frequencies (220–230 kHz, ExAblate Neuro) favor deeper penetration through the intact skull and are suited to targets distributed across the cerebral parenchyma, whereas higher frequencies (500 kHz, NaviFUS) may offer improved spatial resolution and targeting precision for more superficial lesions, with the additional clinical advantage of frameless neuronavigation-guided positioning that eliminates the need for a stereotactic frame. Whether operating frequency differences materially influence the magnitude, spatial distribution, or reversibility of BBBO in the clinical setting remains to be systematically investigated. Agreed reporting standards for FUS-BBBO clinical studies, analogous to CONSORT or TREND guidelines for interventional trials, are needed to enable meaningful cross-study comparisons and accelerate clinical translation.

### 4.6. Limitations

This review has several limitations. First, the total evidence base remains small, with studies characterized by limited sample sizes and heterogeneous designs, which precludes subgroup analyses by tumor grade, IDH status, FUS platform, or drug regimen and limits the generalizability of the findings. Second, the substantial heterogeneity in study designs, patient populations, FUS parameters, and outcome reporting prevented formal meta-analysis; all syntheses are therefore qualitative in nature. Third, IDH mutation status, which is a prognostically critical variable in glioma classification, was not systematically reported across studies, limiting molecular stratification of the available evidence. Fourth, no included study reported quality of life or patient-reported outcomes, leaving an important patient-centred dimension of the treatment experience uncharacterized.

### 4.7. Future Directions

The immediate priority is a Phase III RCT with prospective stratification by MGMT methylation status, IDH mutation, and extent of resection, incorporating patient-reported outcomes. Beyond TMZ, FUS-BBBO offers a delivery platform for therapeutics currently limited by poor CNS penetration, particularly immune checkpoint inhibitors, whose large molecular weight restricts BBB crossing and whose efficacy in GBM may be further enhanced by the immunomodulatory effects of BBBO itself. The sono-liquid biopsy application demonstrated by Woodworth et al. [26], enabling enrichment of circulating tumor DNA post-BBBO, represents a promising parallel avenue for non-invasive tumor monitoring that warrants prospective validation. Finally, the extension of FUS-BBBO to WHO grades II–III IDH-mutant gliomas, where the BBB is uniformly intact and longer natural history allows sustained multi-cycle protocols, remains an underexplored but scientifically compelling direction.

Beyond conventional chemotherapy, FUS-BBBO may facilitate the delivery of novel intracranial agents and synergize with advanced radiation therapy strategies, including stereotactic re-irradiation and radiosensitizer delivery, offering a multimodal approach to tumor control in recurrent and treatment-refractory disease [34].

## 5. Conclusions

FUS-mediated BBB opening represents an increasingly well-characterized and reproducible approach to enhancing intratumoral drug delivery in adult diffuse gliomas. Across the clinical evidence reviewed here, successful BBB opening was achieved in all studies, with a consistent and favorable safety profile—no procedure-related serious adverse events were attributable to FUS-BBBO in any of the seven cohorts examined. Transient T2*/SWAN imaging findings, though frequent, were uniformly asymptomatic and histologically benign, and their incidence can be further reduced through protocol optimization.

The survival findings reported in the only comparative study to date, a median OS advantage of 12 months and a halving of the hazard of death, provide a preliminary clinical signal of potential benefit that FUS-BBBO may translate into meaningful oncological benefit when integrated with standard adjuvant chemotherapy in newly diagnosed GBM. While the non-randomized design of this study requires cautious interpretation, it supports further prospective evaluation in adequately powered randomized controlled trials.

Looking ahead, FUS-BBBO holds promise not only as a chemotherapy delivery enhancer, but as a platform technology for CNS drug delivery more broadly—encompassing immunotherapeutics, targeted agents, and emerging biological therapies currently excluded from the glioma treatment armamentarium by the BBB. The parallel application of sono-liquid biopsy for non-invasive tumor monitoring adds a further dimension of clinical utility. Realizing this potential will require the standardization of technical protocols, prospective molecular stratification in future trials, and the systematic reporting of quality of life and patient-centred outcomes—dimensions that remain conspicuously absent from the current literature.

## Figures and Tables

**Figure 1:**
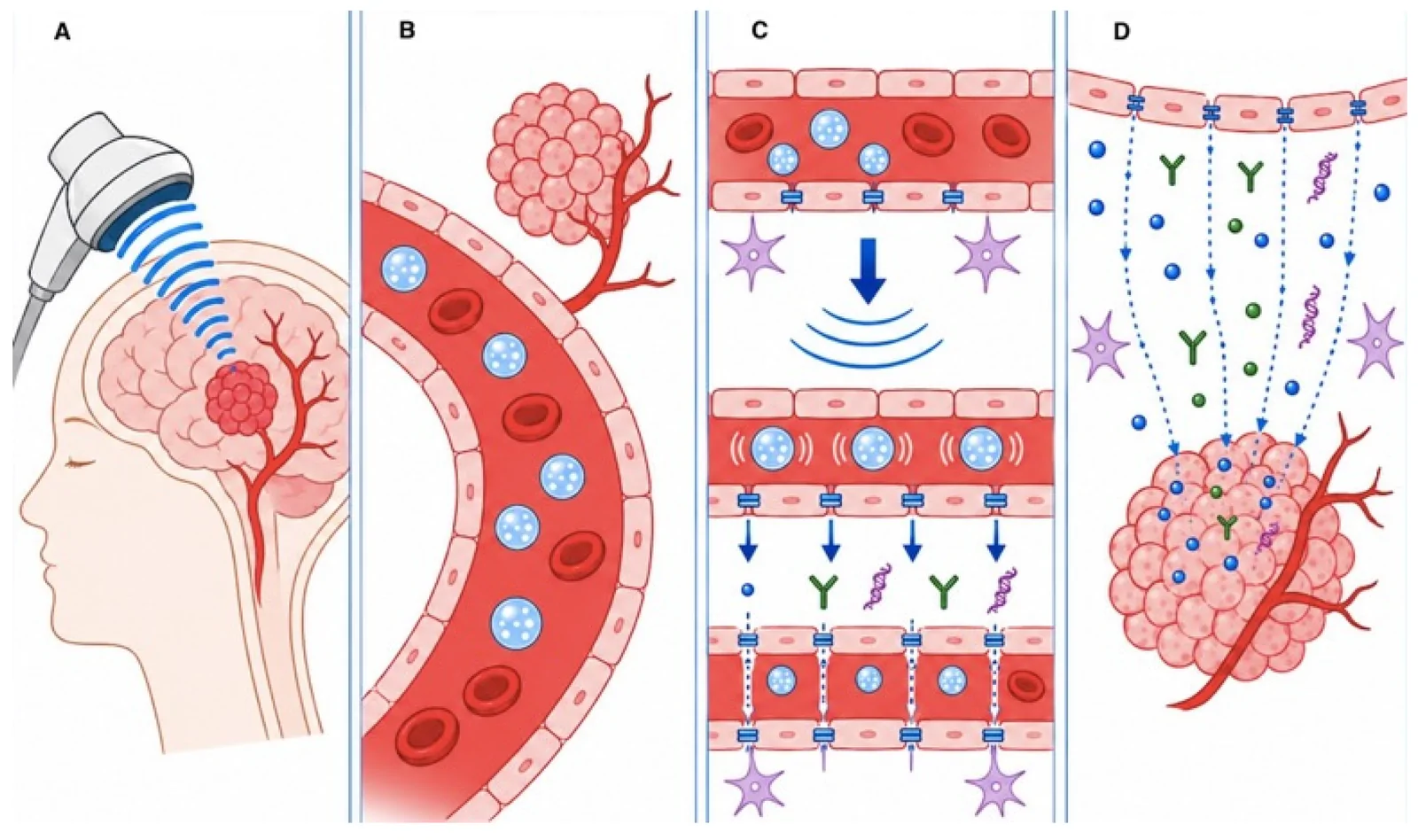
**Schematic overview of focused ultrasound (FUS)-mediated blood–brain barrier (BBB) opening for enhanced delivery to glioblastoma**. (**A**) Transcranial FUS is focused on the tumor region. (**B**) Intravenously administered microbubbles circulate through tumor-associated vessels. (**C**) The interaction between FUS and microbubbles induces a transient, localized, and reversible opening of endothelial tight junctions. (**D**) The resulting increase in BBB permeability facilitates the delivery of therapeutic agents, including chemotherapy and immunotherapy, as well as circulating biomarkers, to and from the tumor microenvironment. Created with BioRender.com accessed on 20 April 2026.

**Figure 2:**
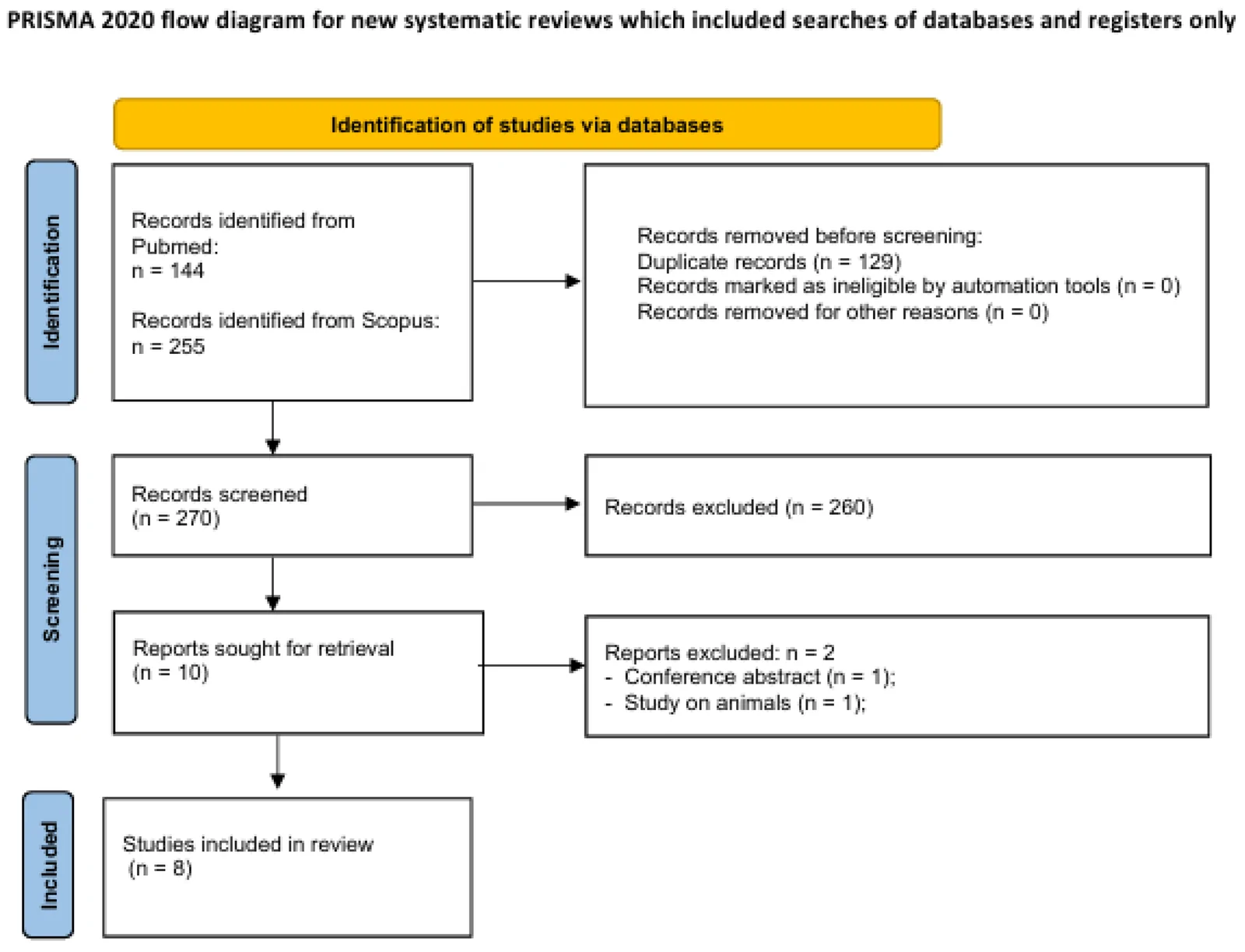
PRISMA flowchart for systematic reviews.

**Table 1:** Characteristics of the included studies and patient demographics.

Author, Year	Design (Phase)	N. Patients	Age (Mean, Range)	Tumor Type	WHO Grade	IDH Status	Treatment Line	FUS System (kHz)	Drug	Sessions (N)
Park et al., [31,32]	Phase I/Pilot +1-year follow-up	6	55.8 ± 11 (n.r.)	GBM	IV (IDH-wt)	IDH-wt (100%)	Adjuvant (post-RT + TMZ)	ExAblate Neuro 220 kHz	TMZ 150–200 mg/m^2^ PO	6 cycles (1 pt: 3 cycles)
Woodworth et al., [26]	Phase II (non-randomized controlled)	34	51.5 ± 13 (n.r.)	HGG (GBM)	III–IV (WHO 2016) [4]	IDH-wt: 85% IDH-mut: 15%	Adjuvant (post-resection + chemoRT)	ExAblate Neuro 220 kHz	TMZ 150 mg/m^2^ PO	Mean 4 cycles (up to 6)
Mainprize et al., [11]	Phase I (pre-surgical)	5	55.8 (33–71)	HGG	III–IV	n.r.	Neoadjuvant (pre-resection)	ExAblate Neuro 220 kHz	TMZ (n = 4) Lipo-DOX (n = 1) PO/i.v.	1 session
Anastasiadis et al., [33]	Phase 0 (pre-surgical)	4	32.5 (29–36)	Infiltrating glioma (LGG/anaplastic)	II–III	IDH-mut (100%)	Neoadjuvant (pre-resection)	ExAblate Neuro 2 230 kHz	Sodium fluorescein 3 mg/kg i.v. (surrogate)	1 session
McDannold et al., [18]	Retrospective technical analysis (BT008 substudy)	9	n.r.	GBM (newly diagnosed)	IV	n.r.	Adjuvant (post-chemoRT)	ExAblate Neuro 220 kHz	TMZ (dose n.r.) PO	38 sessions (≤6/patient)
Chen et al., [20]	Pilot clinical trial (interim analysis)	6	43.5 (26–57)	rHGG (4 GBM, 1 AOD, 1 AA)	III–IV	n.r.	Recurrent (re-irradiation)	NaviFUS 500 kHz	No chemotherapy (FUS + re-RT only)	24 sessions (4/pt cRT; 2/pt fSRS)
Del Bene et al., [13]	Technical note/ Case series (BT008 + BT009)	4	55 (45–63)	GBM (2 newly diag. 2 recurrent)	IV	n.r.	Adjuvant TMZ (BT008)/ Recurrent (BT009)	ExAblate Neuro 4000 Type 2 220 kHz	TMZ PO (BT008) Carboplatin i.v. (BT009)	20 sessions (3 pts: 6 cycles; 1 pt: 2 cycles)

Abbreviations: AA = anaplastic astrocytoma; AOD = anaplastic oligodendroglioma; chemoRT = chemoradiotherapy; DOX = liposomal doxorubicin; FUS = focused ultrasound; fSRS = fractionated stereotactic radiosurgery; GBM = glioblastoma; HGG = high-grade glioma; IDH = isocitrate dehydrogenase; IDH-mut = IDH-mutant; IDH-wt = IDH wild-type; LGG = low-grade glioma; n.r. = not reported; PO = per os; PT = patient; rHGG = recurrent HGG; RT = radiotherapy; TMZ = temozolomide.

**Table 2:** FUS technical parameters and blood–brain barrier opening outcomes.

Author, Year	FUS System	Freq (kHz)	Acoustic Power	Microbubble Agent	MB Dose	N Sessions (Total)	BBB Opening Verification	Extent of Opening	Target Success (%)	Reversibility
Park et al., [31,32]	ExAblate Neuro (InSightec)	220	n.r.	Definity (perfluorocarbon)	n.r.	6 cycles × 6 pts (1 pt: 3 cycles)	Gd-T1 MRI	Qualitative (new enhancement)	92.4%	Yes (<24 h)
Woodworth et al., [26]	ExAblate Neuro (InSightec)	220	n.r.	Definity (perfluorocarbon)	n.r.	Mean 4 cycles/pt (up to 6)	Gd-T1 MRI	100% of sonications confirmed; 82% of subspot targets	82–100%	n.r.
Mainprize et al., [11]	ExAblate Neuro (InSightec)	220	5–15 W	Definity (perfluorocarbon)	4 µL/kg i.v. bolus	1 session (pre-surgical)	Gd-T1 MRI	15–50% ↑ contrast enhancement signal	n.r.	Yes (~20–24 h)
Anastasiadis et al., [33]	ExAblate Neuro 2 (InSightec)	230	3.4–24.6 W (mean; wide inter-patient range)	Definity (perfluorocarbon)	4–5 µL/kg i.v. bolus	1 session (pre-surgical)	Gd-T1 MRI + AEM (harmonic dose)	New Gd-T1 enhancement in 100% of patients	9–100% (per subspot; variable)	Not assessed (direct to surgery)
McDannold et al., [18]	ExAblate Neuro (InSightec)	220	10–30 W (acoustic power; evolved over study)	Definity (perfluorocarbon)	0.24 µL/kg/min i.v. continuous infusion	38 sessions (9 pts; ≤6/pt)	Gd-T1 MRI + SWAN (T2*)	BBBD confirmed in 8/9 patients; correlated with cavitation dose	0–100% (per subspot; highly variable)	n.r.
Chen et al., [20]	NaviFUS (NaviFUS Corp.)	500	0.53–0.61 MI (mechanical index)	SonoVue (sulphur hexafluoride)	0.1 mL/kg i.v. bolus	24 sessions (6 pts total; 4/pt cRT; 2/pt fSRS)	CE-MRI (T1 + T2)	BBBO confirmed; not quantified	n.r.	n.r.
Del Bene et al., [13]	ExAblate Neuro 4000 Type 2 (InSightec)	220	30 ± 10 W (average acoustic power)	Definity/microbubble resonators	n.r.	20 sessions (3 pts: 6 cycles; 1 pt: 2 cycles)	Gd-T1 MRI + SWAN (in-transducer protocol)	~20.5 ± 4.63 cc treated volume; dotted T1 enhancement + SWAN foci	n.r.	Yes (<24 h T1; T2* slower)

**Abbreviations:** AEM = acoustic emissions monitoring; BBB = blood–brain barrier; BBBO = BBB opening; CE = contrast enhancement; FUS = focused ultrasound; Gd = gadolinium;; MB = microbubble; MRI = magnetic resonance imaging; n.r. = not reported; SWAN = susceptibility-weighted angiography; T1 = T1-weighted MRI; T2* = T2*-weighted MRI.

**Table 3:** Drug delivery efficacy and clinical outcomes.

Author, Year	Therapy	Dose/Route	Timing vs. FUS	Intratumoral Drug Conc.	Radiological Response (RANO)	Median OS	Median PFS	Liquid Biopsy
Park et al., [31,32]	TMZ	150–200 mg/m^2^ PO	Day 1 or 2 of each TMZ cycle (post-FUS)	Not measured	Stable disease in all patients during active treatment	OS at 1 year: 100% (2 recurrences at 11 and 16 months)	n.r. (mean time to recurrence: ~13.5 months)	No
Woodworth et al., [26]	TMZ	150 mg/m^2^ PO	Day 1, 2, or 3 of each monthly cycle (post-FUS)	Not measured (indirect inference from survival benefit)	Improved vs. controls (formal RANO n.r.)	31.3 months vs. 19.3 months (controls) HR 0.50 *p* = 0.009	13.5 months vs. 8.1 months (controls) HR 0.55 *p* = 0.020	Yes (cfDNA enrichment post-BBBO)
Mainprize et al., [11]	TMZ (n = 4) Lipo-DOX (n = 1)	TMZ: 140–160 mg PO DOX: 58 mg i.v.	1 h before sonication (pre-FUS)	Higher in sonicated vs. non-sonicated tissue (LC-MS; 2 patients; not quantified statistically)	Not assessed (pre-surgical)	n.r.	n.r.	No
Anastasiadis et al., [33]	Sodium fluorescein (surrogate marker; not therapeutic)	3 mg/kg i.v.	2.5–3.5 h after FUS (pre-surgical incision)	↑ 2.2-fold in FUS-treated vs. untreated tissue (*p* < 0.01; CD31-normalised)	Not assessed (pre-surgical)	n.r. (median FU 15.7 months; 0 recurrences at publication)	n.r.	No
McDannold et al., [18]	Temozolomide (TMZ)	Dose n.r. PO	Immediately pre-TMZ (day 1, 2, or 3)	Not measured	n.r.	n.r. (OS/PFS in	n.r. (OS/PFS in	No
Chen et al., [20]	No chemotherapy (FUS + re-RT only)	Re-RT: cRT 30–40 Gy or fSRS 21–27 Gy	FUS within 2 h before each RT fraction	Not applicable (no drug administered)	ORR 16.7% (1 PR, 2 SD, 3 PD) 6 patients	n.r.	Median 97.5 days	No
Del Bene et al., [13]	TMZ (BT008) Carboplatin (BT009)	TMZ: 150–200 mg/m^2^ PO Carbo: Calvert formula i.v.	TMZ: within 3 h Carbo: within 1 h of BBBO	Not measured	n.r.	n.r. (OS/PFS in for BT008)	n.r.	No

Abbreviations: PO = per os; i.v. = Intravenous; ORR = Objective Response Rate; PFS = Progression-Free Survival; n.r = Not Reported; SD = Standard Dose; re-RT = re-radiotherapy; PD = Progressive Disease.

**Table 4:** Safety profile across the included studies.

Author, Year	Sessions (N Total)	AEs CTCAE Grade ≥ 3	T2*/SWAN Findings	Other Aes (Any Grade)	Attribution	Resolution
Park et al., [31,32]	6 cycles (5 pts) 3 cycles (1 pt)	0 FUS-related 1 CTCAE gr II haematological (patient 6; not FUS-related)	None reported	None reported	All Aes attributed to TMZ or underlying disease; none to FUS-BBBO	Full resolution; no long-term sequelae
Woodworth et al., [26]	Mean 4 cycles/pt (up to 6; 34 patients)	13 grade 3–5 events total (across 34 pts) 0 FUS-related	No symptomatic haemorrhages reported	Consistent with adjuvant TMZ toxicity profile and GBM natural history	Grade 3–5 Aes attributed to TMZ or disease; none to FUS-BBBO	n.r.
Mainprize et al., [11]	1 session (5 patients)	0 FUS-related 1 surgical complication (gr III hemianopia; not FUS-related)	Negative for microhaemorrhages on T2* (1 patient assessed)	Mild headache at helmet attachment sites (2 patients; resolved pre-op)	Hemianopia: surgical (temporal artery sacrifice); not FUS. Headache: FUS procedure-related (minor)	Hemianopia resolved by post-op day 14. Headache: resolved before surgery
Anastasiadis et al., [33]	1 session (4 patients)	0	New GRE/T2* signal in ~50% of targeted subspots Histology: no microhaemorrhages	0 neurological Aes 0 clinical deterioration	GRE/T2* changes: not haemorrhagic (histologically confirmed in resected tissue)	T2* changes assessed at surgery (2–4 h post-FUS); not followed long-term
McDannold et al., [18]	38 sessions (9 patients)	0 FUS-related	SWAN petechiae in all 9 patients (≥1 session) 2 patients: sulcal blood accumulation (asymptomatic)	Nausea in 1 patient (1 session; aborted)	Petechiae: likely micro- leakage/venular dilation; Sulcal blood: asymptomatic, self-limiting	Most petechiae resolved within 1 month. Sulcal blood: resolved before next session
Chen et al., [20]	24 sessions (6 patients)	0 FUS-related 1 gr III radiation necrosis (re-RT related)	None reported	0 FUS-related Aes across all 24 sessions	Radiation necrosis: re-RT related (not FUS). No FUS-BBBO AE identified	Radiation necrosis managed with BEV + re-operation; residual neurological deficit
Del Bene et al., [13]	20 sessions (4 patients)	0	Transient SWAN/ T2* hypointense foci (all patients) Histology (BT008): no microhaemorrhages	0 clinical Aes reported	T2* foci: interpreted as microleakage or venular dilatation; not haemorrhagic (histologically confirmed)	T1 enhancement: resolved <24 h. T2* foci: slower resolution (weeks); confirmed benign

Abbreviations: AE = adverse event; BEV = bevacizumab; CTCAE = Common Terminology Criteria for Adverse Events; FUS = focused ultrasound; BBBO = blood–brain barrier opening; GRE = gradient echo; n.r. = not reported; RT = radiotherapy; SWAN = susceptibility-weighted angiography; T2* = T2*-weighted MRI; TMZ = temozolomide.

## Data Availability

No new data were created or analyzed in this study. Data sharing is not applicable to this article.

## Supplementary Materials

The following supporting information can be downloaded at: https://www.mdpi.com/article/10.3390/biomedicines14102251/s1, Table S1: Prisma-SR checklist; Figure S1: NIH Tool for risk of bias; Figure S2: Robins-I tool for risk of bias.

## Abbreviations

The following abbreviations are used in this manuscript:

AA	Anaplastic astrocytoma
AEM	Acoustic emissions monitoring
AOD	Anaplastic oligodendroglioma
BBB	Blood–brain barrier
BBBO	Blood–brain barrier opening
BBBD	Blood–brain barrier disruption
BTB	Blood–tumour barrier
CE	Contrast enhancement
cfDNA	Circulating tumour DNA
CNS	Central nervous system
cRT	Conventional radiotherapy
CTCAE	Common Terminology Criteria for Adverse Events
DCE	Dynamic contrast-enhanced (MRI)
DOX	Liposomal doxorubicin
fSRS	Fractionated stereotactic radiosurgery
FUS	Focused ultrasound
Gd	Gadolinium
GBM	Glioblastoma multiforme
GRE	Gradient echo (MRI)
HD	Harmonic dose
HGG	High-grade glioma
HR	Hazard ratio
IDH	Isocitrate dehydrogenase
IDH-mut	IDH-mutant
IDH-wt	IDH wild-type
LC-MS	Liquid chromatography-mass spectrometry
LGG	Low-grade glioma
MB	Microbubble
MB-FUS	Microbubble-enhanced focused ultrasound
MGMT	O6-methylguanine-DNA methyltransferase
MI	Mechanical index
MRI	Magnetic resonance imaging
MRgFUS	MRI-guided focused ultrasound
n.r.	Not reported
NIH	National Institutes of Health
ORR	Objective response rate
OS	Overall survival
PD	Progressive disease
PFS	Progression-free survival
PICO	Population, Intervention, Comparator, Outcomes
PO	Per os (oral)
PR	Partial response
PRISMA	Preferred Reporting Items for Systematic Reviews and Meta-Analyses
RANO	Response Assessment in Neuro-Oncology
RCT	Randomised controlled trial
rHGG	Recurrent high-grade glioma
ROBINS-I	Risk Of Bias In Non-randomised Studies of Interventions
RT	Radiotherapy
SD	Stable disease
SWAN	Susceptibility-weighted angiography
T1	T1—weighted MRI
T2*	T2*—weighted MRI
TMZ	Temozolomide
WHO	World Health Organization
